# Visual Outcomes and Prognostic Factors of Intralenticular Foreign Bodies in a Tertiary Hospital in North China

**DOI:** 10.1155/2019/4964595

**Published:** 2019-10-13

**Authors:** Shaolei Han, Tingting Wang, Jinchen Jia, Suhuan Sun, Yiming Fan, Guoxing Yang, Zanzhang Yang

**Affiliations:** Department of Ophthalmology, Hebei Provincial Key Laboratory of Ophthalmology, Hebei Eye Hospital, Xingtai, Hebei Province 054000, China

## Abstract

**Purpose:**

The aim of this study is to describe the epidemiological and visual outcomes and to identify the main prognostic factors of intralenticular foreign body (ILFB) injuries.

**Methods:**

We performed a retrospective review of 21 patients (21 eyes) referred to Hebei Eye Hospital in North China from January 2012 to December 2017, who underwent surgical removal of ILFBs and associated ocular trauma repairs. Data regarding the patient demographics, cause of the injury, nature of the ILFB, clinical features, time interval between the injury and the ILFB removal, time interval between the presentation and the surgery, and the initial and final best-corrected visual acuities (BCVAs) were analyzed, and the main prognostic factors were identified.

**Results:**

Male adults were most affected by ILFBs (90.5%). The mean age of the patients was 41.5 years (median: 46 years, range: 21 to 60 years). None of the patients were wearing goggles at the time of the injury. The most common ILFB cause was hammering the metal (57.1%), and most of the ILFBs were metallic (71.4%). After medical treatment, the final BCVA was improved significantly (*Z* = 2.49, *P*=0.015). There was a significant association between the ILFBs with posterior segment injuries and the final BCVA (*χ*^2^ = 10.03, *P*=0.01). Those factors showing no statistical association with the final BCVA included the age (*χ*^2^ = 0.36, *P*=1.0), gender (*χ*^2^ = 0.52, *P*=1.0), nature of the ILFB (*χ*^2^ = 1.11, *P*=0.54), entrance wound location (*χ*^2^ = 2.85, *P*=0.25), and time interval between the injury and the ILFB removal (*χ*^2^ = 1.87, *P*=0.23).

**Conclusion:**

This is the first local study to explore the epidemiology of ILFB injuries and to identify the main prognostic factors. There was a significant association between the ILFBs with posterior segment injuries and the final BCVA. Improved public awareness and strengthened education regarding safety are the key approaches to reduce the incidence of eye injuries.

## 1. Introduction

Open-globe injuries are some of the main causes of severe vision loss leading to blindness worldwide. Intraocular foreign bodies (IOFBs) may be associated with up to 40% of open-globe injuries, especially in terms of work-related injuries [1, 2]. Intralenticular foreign bodies (ILFBs) are a special subgroup of IOFBs. They are not commonly encountered, and they account for approximately 5% to 10% of all IOFBs [3]. The visual acuity of those patients usually shows some decline due to the aggravation of a traumatic cataract. The management of an ILFB includes an assessment of its size, site, material, potential for infection, lenticular damage degree, and the damage degree of other related tissues [4].

As an important cause of preventable blindness, the overall epidemiology, diagnosis, and surgical management of posterior segment IOFBs have been investigated extensively in many countries [5–8]. However, to the best of our knowledge, there have been a limited number of studies regarding ILFBs to date [9, 10]. Therefore, we aimed to describe the epidemiological and clinical features and the visual outcomes of patients with ILFBs in a tertiary hospital in North China. We also aimed to identify the main prognostic factors that affected the visual outcomes, which afford key data to guide the future management of this preventable sight-threatening condition.

## 2. Materials and Methods

This retrospective, interventional case series study collected data from the medical records of all patients with ILFBs treated at Hebei Eye Hospital in North China between January 2012 and December 2017. The study was conducted in accordance with the Declaration of Helsinki, and the study protocol was approved by the Institutional Review Board of Hebei Eye Hospital. Only those patients who underwent surgeries to remove ILFBs with at least 6 months of follow-up were included in this study. Those patients with posterior segment IOFBs, incomplete medical records, a prior history of decreased vision, and a lack of follow-up data were excluded. The follow-ups of all of these patients took place in the outpatient department of the same hospital.

All of the patients underwent detailed trauma histories and complete ophthalmological examinations upon presentation. The ILFB diagnoses were confirmed by an initial clinical examination, a computed tomography imaging scan, or ultrasound biomicroscopy before surgery. In the present study, the ocular injuries were classified according to the Birmingham Eye Trauma Terminology system.

The medical records were traced and individually reviewed. The collected data comprised demographics, cause of the injury, nature of the ILFB, clinical features, time interval between the injury and the ILFB removal, time interval between the presentation and the surgery, and the initial and final best-corrected visual acuities (BCVAs). The initial and final BCVAs were documented based on Snellen acuity charts. The ILFBs were removed through the limbus or a pars plana scleral incision. The magnetic ILFBs were removed by using intraocular foreign body forceps or strong magnets, while the nonmagnetic ones were removed using forceps. The cataract extraction procedure choice (extracapsular cataract extraction, phacoemulsification, or lensectomy) was dependent on the surgeon's experience and the patient's condition. A good visual outcome was determined as a final BCVA equal to or better than 20/200. A poor visual outcome was defined as a final BCVA of less than 20/200.

The statistical analysis of the data was performed using the Statistical Package for the Social Sciences version 18.0 (SPSS Inc., Chicago, IL, USA). Fisher's exact test was used to identify the prognostic factors affecting the final visual outcome, and the Wilcoxon signed-rank test was used to determine whether there was a statistically significant difference between the initial and final BCVAs. A *P* value of less than 0.05 was considered to be statistically significant.

## 3. Results

During the 6-year study period, 265 patients underwent surgeries for the removal of IOFBs; however, 21 (7.9%) patients with ILFBs met the inclusion criteria, and they were included in this retrospective study. The mean age of the patients was 41.5 years old (median: 46 years, range: 21 to 60 years). Of the 21 patients, 19 (90.5%) were males and 2 (9.5%) were females, showing a male predominance. The right eye was injured in 12 of the patients (57.1%), and the left one was injured in 9 of the patients (42.9%). The time interval between the injury and the ILFB removal ranged from the day of the injury to 3 months later (mean: 30.1 days). The average time interval between the presentation and the surgery was 16.1 hours (median: 24 hours, range: 1 to 48 hours). The average follow-up period was 8.3 months (range: 6 months to 1 year). Among the patients, 11 (52.4%) were industrial workers, 6 (28.6%) were farm workers, 2 (9.5%) were drivers, and 2 (9.5%) were coal miners. All of the injuries were sustained during work. In this study, none of the patients had goggles on at the time of the injury.

The most common ILFB cause was hammering the metal (12 patients, 57.1%) followed by using an electric drill (2 patients, 9.5%), chiselling metal (2 patients, 9.5%), broken glasses (2 patients, 9.5%), explosion (2 patients, 9.5%), and tree branch (1 patient, 4.8%). The ILFB causes are summarized in Table 1.

The nature of the ILFB was metal in 15 eyes (15 patients, 71.4%), glass in 2 eyes (2 patients, 9.5%), stone in 2 eyes (2 patients, 9.5%), wood in 1 eye (1 patient, 4.8%), and unknown in 1 eye (1 patient, 4.8%). The natures of the foreign bodies are shown in Table 2. The location of the wound was the central cornea in 8 eyes (8 patients, 38.1%) and the peripheral cornea in 13 eyes (13 patients, 61.9%).

The main ophthalmic manifestations were traumatic cataracts in 20 eyes (20 patients, 95.2%), followed by an iris defect in 13 eyes (13 patients, 61.9%), anterior chamber cells in 13 eyes (13 patients, 61.9%), lens capsule breaches in 4 eyes (4 patients, 19.1%), vitreous haemorrhages in 3 eyes (3 patients, 14.3%), retinal detachments in 2 eyes (2 patients, 9.5%), and endophthalmitis in 2 eyes (2 patients, 9.5%). The clinical features of the patients are described in Table 3.

There were 4 eyes (4 patients) with posterior segment injuries. All of the eyes (100%) underwent endolaser photocoagulation. Either silicone oil (3 eyes, 75%) or gas (1 eye, 25%) was used for the intraocular tamponade. Finally, all of the eyes were left aphakic initially, and the secondary IOL implantations were performed according to the integrity of the lens capsule.

The initial and final BCVAs of the 21 eyes (21 patients) are presented in Table 4. The final BCVA was significantly improved after treatment when compared to the initial BCVA (Wilcoxon signed-rank test: *Z* = 2.49, *P*=0.013).

There was a significant association between the ILFBs with posterior segment injuries and the final BCVA (Fisher's exact test: *χ*^2^ = 10.03, *P*=0.01). Those factors showing no statistical association with the final BCVA included the age (Fisher's exact test: *χ*^2^ = 0.36, *P*=1.0), gender (Fisher's exact test: *χ*^2^ = 0.52, *P*=1.0), nature of the ILFB (Fisher's exact test: *χ*^2^ = 1.11, *P*=0.54), entrance wound location (Fisher's exact test: *χ*^2^ = 2.85, *P*=0.25), and time interval between the injury and the ILFB removal (Fisher's exact test: *χ*^2^ = 1.87, *P*=0.23) (Table 5).

## 4. Discussion

In this study, we described the epidemiological features, clinical features, and visual outcomes, and we identified the main prognostic factors of 21 patients with ILFB injuries over a 6-year period in North China. In accordance with previous reports [3], the ILFBs constituted 7.9% of all of the IOFBs. Hammering metal was the most common cause for this condition. Thanks to improved surgical techniques and instruments, the final BCVA was significantly improved after medical treatment (*Z* = 2.49, *P*=0.013).

It is known that foreign bodies can carry pathogenic bacteria into the eye, doing harm to the vital ocular structures as well as increasing the risk for endophthalmitis. In addition, retained foreign bodies can also cause a toxic reaction and noninfectious inflammation inside the eye. By destroying the lens and abrogating the capsular integrity, the subcapsular epithelium can accelerate the healing of a small breach in the anterior lens capsule by promoting epithelial proliferation, restoring the continuity of the small breach, and restraining the free movement of ions and fluid that may lead to cataract formation. In the current study, there were 20 patients with traumatic cataracts of varying degrees. The initial BCVAs of 14 of the patients were less than 20/200, and surgical treatment was needed. Although the initial BCVAs of 6 of the patients were equal to or better than 20/200, the patients required surgical treatment in order to avoid the occurrence of complications, such as ocular siderosis.

China is the largest developing country in the world. With the implementation of reform and opening up of the country, thousands of factories have been built in China. Because there is no regulatory rule for wearing goggles for safety in our country, many workers are injured when performing high-risk work. Similar to previous studies [11], all of the eye injuries in this study occurred because goggles were not worn, which highlights the importance of goggles for preventing eye injuries. The mean age of the patients was 41.5 years (median: 46 years, range: 21 to 60 years). The fact that most of the patients were males and within the working age, the range is consistent with the literature [6], which suggests that young to middle-aged working males are more likely to suffer from ocular trauma. The present study also revealed that hammering metal was the most common cause of injury, which is similar to that of most other studies [12]. However, this situation is different from that in Thailand, where these injuries are often caused by electric grass trimmers [13].

The natural course of a retained ILFB varies widely. Small ILFBs may be completely resorbed, become encapsulated, or lose their magnetic properties [1, 14–16]. In the present study, the foreign bodies were metal in 15 eyes (15 patients, 71.4%), glass in 2 eyes (2 patients, 9.5%), stone in 2 eyes (2 patients, 9.5%), wood in 1 eye (1 patient, 4.8%), and unknown in 1 eye (1 patient, 4.8%). We could not find a statistical association between the nature of the foreign body and the final BCVA (*χ*^2^ = 1.11, *P*=0.54). It has been reported [17–19] that the development of ocular siderosis is generally considered to be the most serious complication of iron retention with foreign bodies, and this can cause persistent iron toxicity to the retina and other intraocular tissues for a long time.

The cornea possesses most of the exposed area of the ocular surface; therefore, it is the location most likely to suffer an ILFB entrance wound. Moreover, a wound that occurs in the central cornea is likely to have a worse result than that in the peripheral cornea. However, in the present study, the entrance wound location was limited to within the central cornea in 8 eyes (38.1%) and to within the peripheral cornea in 13 eyes (61.9%). Moreover, there was no statistical association between the entrance wound location and the final BCVA (*χ*^2^ = 2.85, *P*=0.25); similar results have been published by Erakgun and Egrilmez [20]. One possible reason is that the presence of a corneal scar can give rise to high astigmatism, which further aggravates the visual impairment, leading to a significantly reduced initial BCVA. However, with the development of better surgical technology, these abnormalities can be successfully treated via surgery, and they will not affect the final BCVA.

The time interval between the injury and the ILFB removal is also important. To rescue visual function and to avoid further damage and the severe complications caused by retained ILFBs, an extraction of the ILFB should be performed as soon as the diagnosis is made. Early ILFB removal can lessen the risk of complications directly related to ILFBs, and it might decrease the incidence of acute post-traumatic endophthalmitis. Some studies have shown that [11] the shortest interval between the trauma and the foreign body removal was a significant predictor for a good final BCVA. In the present study, the time interval between the injury and the ILFB removal was less than 24 hours in 5 eyes (23.8%) and more than 24 hours in 16 eyes (76.2%). However, there was no statistical association (*χ*^2^ = 1.87, *P*=0.23) between the intervention time and the final BCVA. One reason is that it is possible that patients with ILFB injuries are more likely to receive early and active treatment. Another possible reason may be that the injury induced by an ILFB is often limited to within the anterior segment, where the lens acts as a protective barrier. Finally, when a high-velocity projectile enters the eye, it may self-sterilize due to the heat generated by friction.

In previous studies [21–23], posterior segment IOFBs were usually associated with more complicated conditions, such as retinal detachment, endophthalmitis, proliferative vitreoretinopathy, and an epiretinal membrane, and they were severely damaged, with a relatively poor final BCVA. An anterior IOFB usually has a better final BCVA than a posterior IOFB. However, in this study, 4 patients with posterior segment injuries present before surgery, such as a vitreous haemorrhage, retinal detachment, and endophthalmitis, had relatively poor final BCVAs. There was a significant association between the ILFBs with posterior segment injuries and the visual outcomes (*χ*^2^ = 10.03, *P*=0.01). Moreover, the recovery of the BCVA was dependent mainly on the condition of the posterior segment.

We acknowledge that our study had its share of limitations. The first and foremost is related to its retrospective nature. Second, our sample size was not large enough to allow for accurate statistical calculations for investigating the factors associated with the outcomes. In the future, a randomized prospective clinical trial will be required to determine other prognostic factors that can be used to predict visual outcomes. Nevertheless, our study provided epidemiological information about ILFBs in our locality. These results are important parameters that can be used to guide future studies and suggest measures to enhance occupational safety.

## 5. Conclusions

In summary, our study has shown that an ILFB is an important complication of work-related injuries in North China. There was significant association between ILFBs with posterior segment injuries and the final BCVA. As ophthalmologists, we want to stress to our patients that improved public awareness and strengthened safety education are the key approaches to reduce the incidence of eye injuries.

## Figures and Tables

**Table 1:** Cause of the injury.

Cause of injury	Number (%)
Hammering the metal	12 (57.1%)
Using electric drill	2 (9.5%)
Chiselling metal	2 (9.5%)
Broken glasses	2 (9.5%)
Explosion	2 (9.5%)
Tree branch	1 (4.8%)

**Table 2:** Nature of the ILFB.

Nature of the ILFB	Number (%)
Metal	15 (71.4%)
Glass	2 (9.5%)
Stone	2 (9.5%)
Wood	1 (4.8%)
Unknown	1 (4.8%)

**Table 3:** Clinical features of the patients.

Clinical features	Number (%)
Traumatic cataracts	20 (95.2%)
Iris defects	13 (61.9%)
Anterior chamber cells	13 (61.9%)
Lens capsule breaches	4 (19.1%)
Vitreous haemorrhages	3 (14.3%)
Retinal detachment	2 (9.5%)
Endophthalmitis	2 (9.5%)

**Table 4:** The initial and final BCVA.

Category	The initial BCVA number (%)	The final BCVA number (%)	*Z* value	*P* value
NLP-HM	5 (23.8%)	0 (0%)		
CF-0.1	8 (38.1%)	4 (19.1%)		
0.12–0.3	1 (4.8%)	5 (23.8%)		
0.3–1.0	7 (33.3%)	12 (57.1%)		
Total eyes	21 (100%)	21 (100%)	2.49	0.013

NLP: no light perception; HM: hand motions; CF: counting fingers.

**Table 5:** Factors that may influence the final visual outcome.

Factors	BCVA <0.1	BCVA ≥0.1	*χ* ^2^ value	*P* value
	*n* = 4 (19.1%)	*n* = 17 (80.9%)		
Age (years)			0.36	1.0
≤40	1 (4.8%)	7 (33.3%)		
>40	3 (14.3%)	10 (47.6%)		
Gender			0.52	1.0
Male	4 (19.1%)	15 (71.4%)		
Female	0 (0%)	2 (9.5%)		
Nature of ILFB			1.11	0.54
Metal	2 (9.5%)	13 (61.9%)		
No metal	2 (9.5%)	4 (19.1%)		
Wound location			2.85	0.25
Central cornea	3 (14.3%)	5 (23.8%)		
Peripheral cornea	1 (4.8%)	12 (57.1%)		
Interval time between the injury and the ILFB removal			1.87	0.23
≤24 h	2 (9.5%)	3 (14.3%)		
>24 h	2 (9.5%)	14 (66.7%)		
Posterior segment injuries			10.03	0.01
Yes	3 (14.3%)	1 (4.8%)		
No	1 (4.8%)	16 (76.2%)		

## Data Availability

The data used to support the finding of this study are available from the corresponding author upon reasonable request.
